# Complete Resolution of a Large Bicuspid Aortic Valve Thrombus with Anticoagulation in Primary Antiphospholipid Syndrome

**DOI:** 10.3389/fcvm.2017.00059

**Published:** 2017-09-20

**Authors:** Rayan Jo Rachwan, Ghassan E. Daher, Jawad Fares, Rachoin Rachoin

**Affiliations:** ^1^Department of Medicine, Indiana University School of Medicine, Indianapolis, IN, United States; ^2^Department of Internal Medicine, Saint Louis University School of Medicine, St. Louis, MO, United States; ^3^Faculty of Medicine, American University of Beirut, Beirut, Lebanon; ^4^Division of Cardiovascular Medicine, Notre Dame des Secours University Hospital, Byblos, Lebanon

**Keywords:** antiphospholipid syndrome, aortic valve, thrombosis, anticoagulation, echocardiography

## Abstract

Native aortic valve thrombosis in primary antiphospholipid syndrome (APLS) is a rare entity. We describe a 38-year-old man who presented with neurological symptoms and a cardiac murmur. Transthoracic echocardiography detected a large bicuspid aortic valve thrombus. Laboratory evaluation showed the presence of antiphospholipid antibodies. Anticoagulation was started, and serial echocardiographic studies showed complete resolution of the aortic valve vegetation after 4 months. The patient improved clinically and had no residual symptoms. This report and review of the literature suggests that vegetations in APLS can be treated successfully with conservative treatment, regardless of their size.

## Introduction

Antiphospholipid syndrome (APLS) is a systemic autoimmune disorder characterized by the presence of antiphospholipid antibodies (aPLs) and clinical features, mainly arterial and/or venous thrombosis and/or fetal loss. APLS can be classified as primary in the absence of another autoimmune disease, or as secondary in the presence of an underlying disorder, most commonly systemic lupus erythematosus. aPLs have been found in around 5% of the general population (1); however, only a small proportion will develop APLS. APLS has been estimated to have an incidence of 5 new cases per 100,000 people per year, and a prevalence of 40–50 cases per 100,000 people per year (2). According to the Sydney criteria (3), APLS is diagnosed based on the presence of at least one clinical event (either a vascular thrombosis and/or adverse obstetric event), and the presence of aPL [either anticardiolipin (aCL), lupus anticoagulant (LA), or anti-β2 glycoprotein-1 (anti-β2GP1)] on two or more occasions, with a minimum 12-week interval. Several clinical features associated with APLS have not been included in the Sydney criteria (3). These features include aPL-associated cardiac valve disease (ACVD), nephropathy, livedo reticularis, and thrombocytopenia.

Antiphospholipid syndrome significantly impacts the cardiovascular system. ACVD, presenting with a valvular mass and/or valvular thickening, is often encountered in APLS. Approximately one-third of patients with primary APLS exhibit ACVD (4). The most commonly affected valve is the mitral valve, followed by the aortic valve (5), with regurgitation being the most common functional abnormality (6). These valvular lesions are usually of minor hemodynamic significance, but have been associated with serious thromboembolic events.

There is no general consensus on the definitive treatment of ACVD. Popular regimens used for the treatment of ACVD include the following: warfarin, antiplatelet agents, and low-molecular-weight heparin (LMWH). The efficacy of anticoagulant therapy on valvular masses is controversial. Some believe that ACVD valvular masses are due to inflammation and thus anticoagulation would be ineffective (7), whereas others were successful with anticoagulation in the treatment of these lesions (8). A small minority of APLS patients (4–6%) develop a valve disease that is severe enough to require valvular surgery (9). However, surgical patients had a higher rate of complications, mostly bleeding and thrombosis (10).

Bicuspid aortic valve (BAV) is the most common congenital heart disease, affecting 1–2% of the population with a higher prevalence (2:1) in males (11). Individuals with BAV have a potential risk of complications; the most commonly being aortic stenosis, aortic regurgitation, aortic dissection, and infective endocarditis (IE). Aortic valve thrombosis in the setting of BAV is a rare complication, and only few cases have been reported (12).

To the best of our knowledge, complete resolution of a large bicuspid aortic mass with anticoagulation in the setting of APLS has not been reported in the medical literature. Therefore, this communication explores this rare phenomenon with a review of the literature.

## Case Report

A 38-year-old man was referred to us, by his primary care physician, for evaluation of possible aortic valvulopathy. He is known to have dyslipidemia; for which he was not taking any medications. He is a heavy smoker (45 pack-year), drinks alcohol occasionally, and denies drug-use.

Six months before presentation, he started having short episodes (<10 min) of left-arm numbness and weakness with headache and dizziness. He also reported having dyspnea upon exertion. One month prior to his presentation, the patient was hospitalized due to the exacerbation of his clinical symptoms, in addition to a 2-h episode of ataxia and diplopia. Initial workup included computed tomography (CT) and magnetic resonance imaging of the brain, electroencephalogram, and lumbar puncture; all of which were unremarkable. Patient was suspected to have simple partial seizure and atypical migraine, and was discharged on carbamazepine and prophylactic propranolol. Upon follow up with his primary care physician, the patient’s symptoms did not improve and a thorough physical examination revealed a cardiac murmur in the aortic region. Based on this new cardiac finding, the patient was referred to us for further evaluation and management.

Upon presentation to our clinic, he was afebrile and hemodynamically stable. Cardiovascular examination revealed a combined systolic–diastolic murmur best heard at the second right intercostal space, suggesting aortic valve disease. The rest of the physical examination was unremarkable.

**Figure 1:**
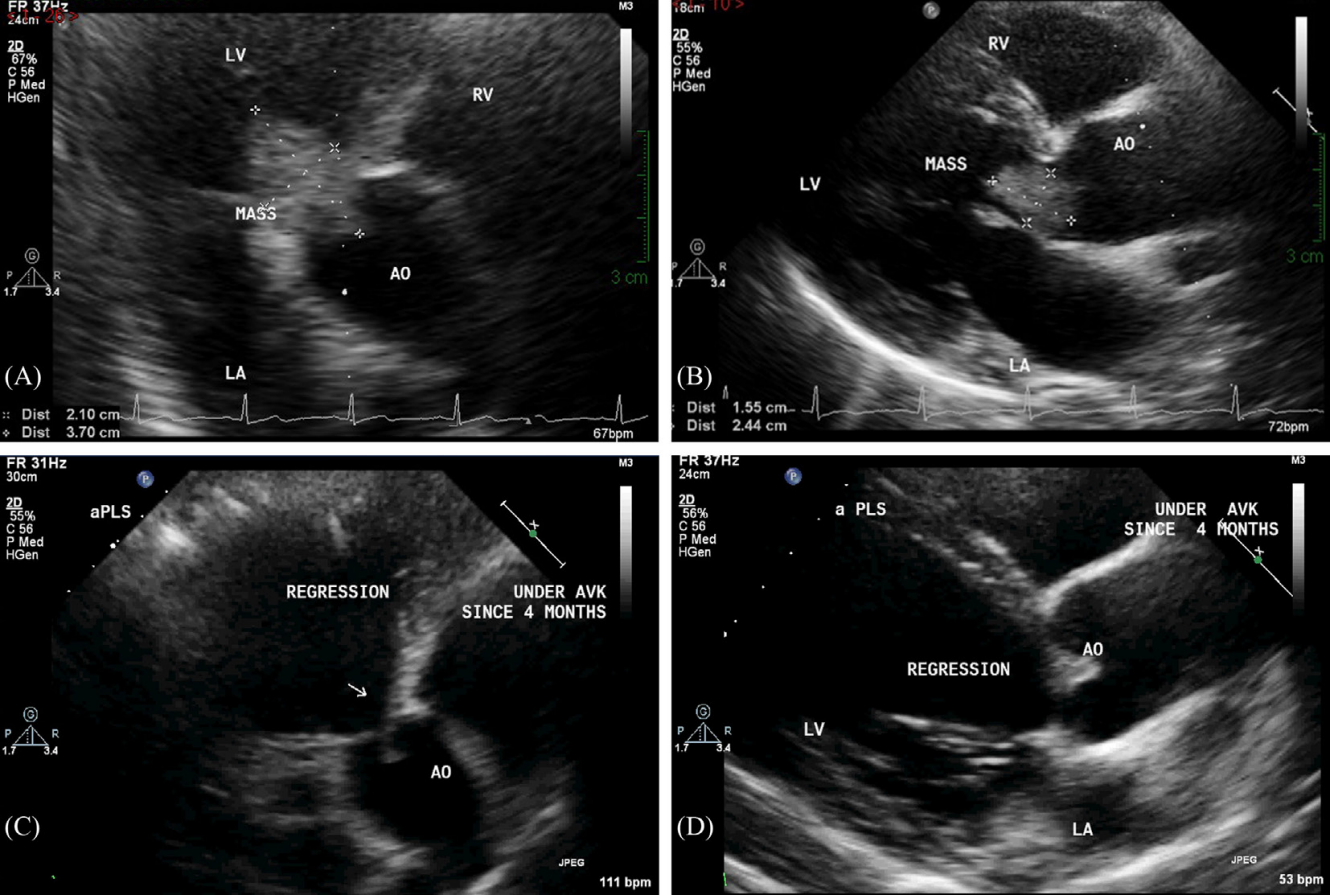
Transthoracic echocardiography performed at presentation reveals a large ovoid laminated mass on the aortic valve, measuring 3.7 cm × 2.1 cm on apical five chamber view **(A)** and 2.4 cm × 1.6 cm on parasternal short-axis view **(B)**. Follow-up transthoracic echocardiography performed after 4 months of anticoagulation shows complete resolution of the valvular mass on apical five-chamber view **(C)** and parasternal short-axis view **(D)**.

An electrocardiogram, done at presentation, revealed left ventricular hypertrophy. A transthoracic echocardiogram (TTE) showed an irregular ovoid laminated mass, 3.7 cm × 2.1 cm in size (Figures 1A,B). The mass was firmly attached to the aortic valve surface and exhibited no independent motion. Doppler echocardiography revealed Grade II–III aortic regurgitation and a mean gradient of 21 mmHg across the aortic valve. There was also evidence of moderate left ventricular hypertrophy and dilation with a normal ejection fraction (>55%).

Patient was then admitted to the hospital for further workup of his condition. Laboratory studies revealed a normal complete blood count, an erythrocyte sedimentation rate of 64 mm/h, a C-reactive protein of 20 mg/L, and negative blood cultures (three separate sets). Cardiac enzymes were normal and chest radiography showed no significant findings.

Hypercoagulability workup was done. It revealed the presence of aCL (IgG isotype) in serum with a titer of 205 GPL (normal level < 20 GPL) and was positive for LA; anti-β2GP1 was not tested for technical reasons. The levels of protein C, protein S, factor V Leiden, and homocysteine were normal. Serological markers for connective tissue disorders, including antinuclear antibodies, rheumatoid factor, anti-neutrophilic–cytoplasmic antibodies, anti-double-stranded-DNA antibodies, and anti-Smith antibodies were all negative. Serologies for hepatitis B, C, and HIV were negative. Furthermore, CT scans of the chest, abdomen, and pelvis were insignificant.

The diagnosis of non-bacterial thrombotic endocarditis (NBTE) in the setting of primary APLS was suspected. The patient was started on anticoagulation with LMWH and then bridged to oral warfarin; INR 2–3 was maintained. Carbamazepine and propranolol were discontinued. The patient was educated about the importance of smoking cessation and adherence to his statin therapy.

**Figure 2:**
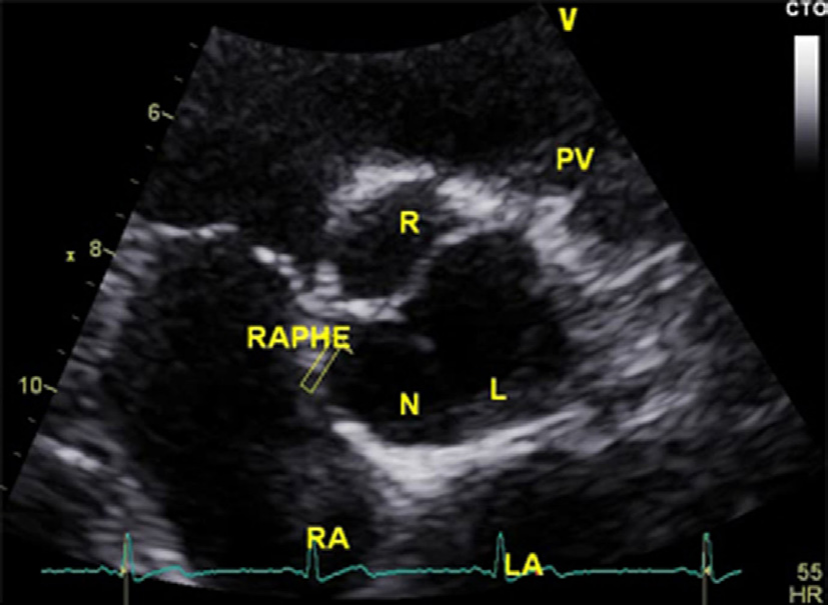
Follow-up transthoracic echocardiography performed 5 months after presentation, showing the presence of previously undiagnosed bicuspid aortic valve.

At the 3-month follow-up, only aCL level was repeated and was found to be elevated (170 GPL). Serial TTE controls showed progressive resolution of the mass, with complete regression 4 months after therapy (Figures 1C,D). In addition, there was regression in the aortic regurgitation to Grade I, and reduction in the left ventricular hypertrophy and dilation. Interestingly, the resolution of the valvular mass unveiled a BAV that was not previously diagnosed (Figure 2).

Furthermore, a cardiac CT was done to rule out APLS-induced coronary artery disease; it showed no abnormalities. The patient was maintained on warfarin, and subsequent follow-ups showed him to be clinically asymptomatic.

## Discussion

Cerebral involvement is prominent in primary APLS; with stroke (19.8%) and transient ischemic attack (TIA) (11.1%) being its most common clinical manifestations (13). Hughes et al. (14) reported that in young patients (<45 years), more than 20% of strokes are potentially associated with APLS. Recurrent transient episodes of visual disturbances, numbness, weakness, and dizziness can all be expressions of TIA (15). All these were present in our patient and might explain his symptoms.

Arterial thrombosis involves the brain vasculature in more than 50% of the cases, and it is the main cause of cerebral ischemia in primary APLS (16). However, an association has been reported between ACVD and central nervous system manifestations of the syndrome, which suggests that cerebral emboli from non-infectious valvular lesions, often referred to as NBTE, may be a risk factor (17).

Valvular masses have a wide differential diagnosis, which includes NBTE, IE, and cardiac tumor. It is clinically challenging to distinguish between IE and NBTE due to APLS. In fact, both share many clinical features including vascular thromboembolic events, valvular vegetations, and renal and cutaneous involvement. In addition, fever can be present in APLS, and aPL can be frequently found to be temporarily elevated during infections (18). Our patient did not satisfy the modified Duke criteria for IE. Since these criteria are sensitive for disease detection and have a high negative predictive value (19, 20), the diagnosis of IE was rejected.

Aortic valvular masses also raise the suspicion of cardiac tumor, an important differential diagnosis that should not be overlooked. The best way to diagnose a cardiac tumor is by excision and histopathologic examination. However, echocardiography can be used to distinguish between a tumor and a thrombus based on imaging characteristics of the mass. Thrombotic mass is characterized by an irregular or lobulated shape, laminated appearance, microcavitations, and absence of a pedicle (21). In contrast, a cardiac tumor usually appears as a small, mobile, pedunculated or sessile valvular, or endocardial mass (22). In our case, the characteristics of the lesion were typical of that of a thrombus.

The Sydney criteria committee proposes a minimal consensus concerning valvular lesions in APLS but argues against adoption as criteria (3). This consensus defines ACVD as the presence of aPL, in addition to echocardiographic detection of a valvular lesion and/or dysfunction (regurgitation and/or stenosis of mitral and/or aortic valve or any combination of the above) (3). In our patient, such features were present and thus swayed our diagnosis toward ACVD manifesting as NBTE.

Echocardiography is essential in the diagnosis of ACVD. About 30–40% of valvular lesions in the setting of APLS can be detected by TTE, while 60–80% of lesions can be detected by transesophageal echocardiography (TEE) (23). TTE can be used initially to detect the presence of a cardiac mass. However, if TTE results were non-diagnostic or equivocal, TEE would be a more accurate modality due to its higher sensitivity and specificity (24).

Native aortic valve thrombosis is a rare event. In ACVD and BAV, valvular dysfunction is associated with abnormal blood flow, which can induce endothelial lesion and trigger thrombus formation (25). Furthermore, coagulopathy in APLS can induce aortic valve thrombosis. This is possibly due to particular affinity of aPL to valve endothelium that leads to formation of an immune complex, which can cause an injury to the endothelium (26). Therefore, we cannot be certain about the exact role that each of ACVD and BAV played in the pathogenesis of the thrombotic mass observed in our case.

In terms of treatment, there have been no set guidelines for the definitive treatment of ACVD. Similar to other reports (27–29), our case has shown anticoagulation to be effective in treating valvular vegetation in primary APLS. The optimal intensity of anticoagulation for the prevention of recurrent thrombosis in patients with APLS is uncertain. Two randomized controlled trials found that high-intensity anticoagulation (INR > 3) was not superior to moderate-intensity anticoagulation (INR 2–3) in patient with APLS, and was associated with a higher rate of bleeding complications (30, 31). Therefore, we suggest the use of anticoagulation with an INR target of 2–3 as a standard of treatment. In addition, most specialists recommend lifelong use of anticoagulation due to the high recurrence rate of thrombotic events in APLS (32, 33). Modification of concomitant risk factors for thrombosis, such as hypertension, dyslipidemia, and smoking cessation, must also be addressed.

In conclusion, our report suggests that conservative treatment with anticoagulation along with vigilant observation might be the best therapeutic plan for patients with aortic valvular masses in the setting of APLS. However, these results should be approached with caution as whether conservative management with anticoagulant or aortic valve replacement ought to be recommended remains unresolved due to the rarity of this condition and the lack of trial data.

## Ethics Statement

This case report was exempted from any ethics committee verification due to its retrospective nature. The echocardiographic images and case presentation were approved by the patient to be used for publication.

## Author Contributions

All authors contributed to the analysis and interpretation of data, wrote the manuscript, approved the final version of the manuscript, and agreed to be accountable for all aspects of the work.

## Conflict of Interest Statement

The authors declare that the research was conducted in the absence of any commercial or financial relationships that could be construed as a potential conflict of interest.
